# A Mass of Interesting Detail

**Published:** 1921-08-13

**Authors:** 


### THE RED CROSS REPORT.

### A Mass of Interesting Detail.

The general report of the Joint War Committees of the British Red Cross Society and the Order of St. John is a formidable-looking document. Consisting of over 800 folio pages, with maps, plans, photographs, and thirty pages of index, it is an encyclopædic record of Red Cross operations from the formation of the first units in 1914 to the distribution of honours at the end of the war. Luckily, the index is so full and the body of the report so well divided, that any particular department of the work can be disengaged. Vast as the report is, it is perhaps worth remembering that the work described was supplementary to that carried on by the Army Medical Service, and that the War Office supplied all "necessaries" for the sick and wounded. The Government's business was much more extensive even than that performed by the Red Cross, and when this fact is remembered we gain some idea of the magnitude of the "business of war" and the complexity of the organisations on which it depended.

Once examined, the report shrinks to readable proportions. We at home may omit, unless our work lay there, parts 16-26, describing the work in different foreign countries. We may note, for permanent reference, the agreements between the two Societies, the rolls of county directors, auxiliary hospitals, and work-parties, and the bibliography of books and reports which make the appendices well worth preserving. Hospitals and their managers are most concerned with the organisation of the Red Cross voluntary effort, in the front of which the raising of the preliminary money is deservedly placed.

The public subscribed £16,500,000 in cash and gave over a million in stores. This is, of course, the largest voluntary effort ever made in this country. Half, or rather more, of the receipts were provided by "Our Day" collections at home and in the Dominions. It is less surprising how the money was spent. Suffice it to remark that 10,000,000 sick and wounded were transported. Now that the war is over, and a lively sense remains of the work accomplished and the means adopted, the details with which the report teems are chiefly valuable as a record and a storehouse of experience should a similar crisis arise once more. The only qualification which must be added is that, while for small wars detailed Red Cross plans made in advance are of great value, in a great war the whole machinery of the nation is so thrown out of gear that peace-made programmes cannot be kept.

Much of the information given is still of present value, such as the work done since demobilisation and the detailed procedure for the organisation of flag days, sales, collections, and the like. To the latter should be added the description of the manner in which "trade collections" were arranged in the seventy trades which possess their own associations. There is, indeed, a mine of suggestion in the whole of Part III., wherein the "sources of income" are described in detail. The sections devoted to the arrangements necessitated by Christie's sales make a fascinating and pregnant chapter, wherein paragraph 88 embodies the lessons learnt, which should be referred to whenever a similar effort on behalf of charity is contemplated. Useful hints upon the law regulating lotteries and the occasions when it is not likely to be enforced are given in connection with the story of the Red Cross pearl necklace, and how the idea of a lottery came to be abandoned. The Societies' experience in charity entertainments is also instructive.

Part XXXIII., entitled the "Conclusion," contains several matters of interest, beginning with the combing out of Red Cross *personnel* and the distribution of honours. It is evident that the Red Cross ambulance service in France could not have been maintained at all if all the category A men had been taken into the Army. Again, the difficulties in the way of a bestowal of honours which shall satisfy all that no decorations were given to those who had not earned them are well described. The desire to avoid injustice, and the fact that a certain number of distinctions were declined, no doubt encouraged the British Red Cross and the Order of St. John to issue their own medals for war service. Beside this, a certificate of thanks has been sent to all who worked under the joint organisation. The spirit permeating these paragraphs on the distribution of honours is also worth remark, because it reveals the personal element which makes the report throughout so human and so interesting. Never, we think, has a mass of information upon voluntary work been better arranged or more interestingly given. Instructive reports often emanate from Government Departments and may bear the signature of the man who has been personally responsible for the matter there recorded. But in all, or almost all, of these we miss the note which informs this voluntary report of an immense voluntary undertaking. Signed by Sir Arthur Stanley and Mr. Evelyn Cecil, the editor appears to have been Mr. Danvers Power. To him, presumably, belongs the credit for a report the alarming size of which may do less justice to its interest. Open it anywhere, and one paragraph leads to another without flagging. The report is not only a record, but a volume of suggestion also.

